# Knowledge on cardiopulmonary resuscitation among rural adults at Bangalore, India

**DOI:** 10.6026/973206300210081

**Published:** 2025-01-31

**Authors:** Ezhilarasi R., Helen Shaji J.C.

**Affiliations:** 1 Department of Nursing Education, Sri Balaji Vidyapeeth (deemed - to be a university), Pondicherry, India; 2 Department of Medical Surgical Nursing SRM College of Nursing, SRMIST, Chennai, Tamilnadu, India

**Keywords:** Cardiopulmonary resuscitation knowledge, rural communities, educational initiatives, emergency readiness, health outcomes

## Abstract

Knowledge of cardiopulmonary resuscitation (CPR) among 253 adults in a rural area of Bangalore is of interest. Data shows a significant knowledge gap, with 81.4% of participants having inadequate understanding of cardiopulmonary resuscitation. Education level emerged as the sole demographic factor significantly associated with cardiopulmonary resuscitation knowledge, highlighting the importance of targeted educational initiatives. Despite prior exposure to cardiopulmonary resuscitation through various channels, widespread misconceptions persisted, emphasizing the need for practical, community-specific training programs. Hence, the need for equipping rural communities with essential life-saving skills to improve pre-hospital emergency care is highlighted.

## Background:

Cardiopulmonary resuscitation (CPR) is a life-saving skill that significantly increases survival rates during cardiac emergencies. Despite its critical importance, cardiopulmonary resuscitation knowledge remains alarmingly low, particularly in rural communities where access to immediate medical assistance is limited. Studies reveal that globally only 2.5% of individuals are trained in cardiopulmonary resuscitation and in India, the percentage is even lower, with Karnataka reporting a rate of 1.5% [1, 2]. This disparity underscores the pressing need for targeted educational initiatives to equip rural populations with essential life-saving skills. Previous research highlights the correlation between cardiopulmonary resuscitation training and improved bystander response during emergencies [3, 4]. However, barriers such as lack of awareness, cultural misconceptions and limited training resources persist, particularly in underserved regions [5, 6]. Therefore, it is it interest to assess knowledge on cardiopulmonary resuscitation among rural adults of Bangalore, India.

## Methodology:

The purpose of the study is to determine the level of knowledge, gaps in knowledge and misconceptions related to cardiopulmonary resuscitation among the surveyed population and to find the association between the demographic variables among adults residing in a selected rural area of Bangalore.

## Hypothesis:

There is a significant relationship between the demographic variables and the knowledge of cardiopulmonary resuscitation of an adult residing in a selected area of Bangalore.

## Novelty:

Many studies have been conducted at both international and national levels. However, very few studies have been conducted in India, particularly in rural areas. Hence, the researcher emphasizes the importance of conducting research at the rural level to enhance knowledge and practice and to create awareness among adults residing in selected areas. Every minute is precious and crucial in an emergency to correct ourselves, provide quality life support and improve lives. This is a cross-sectional study determining cardiopulmonary resuscitation knowledge among Adults residing in the Kempapura village (rural). Around 2650 people lived in Bangalore between February and September 2024.

## Criteria of the study participant:

The study population included both males and females; adults residing in the Kempapura village aged 18 to 25. They were physically and mentally sound and willing to participate in our survey and give their spontaneous opinion regarding their knowledge of cardiopulmonary resuscitation. The participants were selected through a Stratified Random Sampling Technique. This method ensured that different subgroups within the population were adequately represented in the Kempapura village.

## Data collection:

We used self-administered questions as previously described in Kanada and English languages; the questionnaire consisted of Demographic data including Age, Gender, Religion, type of family, size of family, previous knowledge about cardiopulmonary resuscitation (1), self-administered knowledge questionnaire (2) which have 25 questions which can be divided into five sections, general cardiopulmonary resuscitation knowledge (6) questions of practice about cardiopulmonary resuscitation (8), willingness and confidence in performing cardiopulmonary resuscitation in an emergency (5), common misconception and gaps (4), Improvement in the knowledge of cardiopulmonary resuscitation (2), to detect the impression of participants and their suggestions to increase the awareness among the population to determine the validate of our questionnaire with content validity and randomly distributed to survey of 253 adults in this all the 253 questionnaire were appropriately filled with response rate is 100%.

## Data processing and analysis:

The data were analysed using the Statistical Package for Social Sciences version 21. The descriptive, inferential and comparative statistical analyses were performed on the association between the demographic characteristics analysed using the chi-square test and the P value was used to detect the significant parameter on a categorical score between the groups when needed.

## Inclusion criteria:

[1] Residents of selected rural area.

[2] Adults aged 18 years to 40 years.

[3] Consent to participate in the survey.

[4] Those who know how to read and write Kannada.

## Exclusion criteria:

[1] Individuals who are not permanent residents of the selected area.

[2] Individuals below 18 years.

[3] Non consenting individuals.

## Results:

This study was carried out on 253 participants who completed the survey, with a response rate of 100% of the demographic data shown in (Table 1). The distribution of participants that 30.4% (n=77) aged 18-20, 39.1% (n=99) fall within the 21-30 age group and the other 30.4% (n=77) are aged 31-40, a majority of participants are female, making up 66.8% (n=169), while males constitute 33.2% (n=84), most of the participants (47.8) (n=121) have completed primary education, 44.7% (n=113) have higher secondary education. In comparison, 7.1% (n=18) have no formal education-a small fraction of 0.4% (n=1) have undergraduate or post-graduate degrees in the occupation. The largest group of participants are private sector employees, constituting 46.2% (n=117); homemakers represent 39.5% (n=100); unemployed individuals make up 13.0% (n=33), while only 0.4% (n=1) are formers and 0.8% (n=2) are labourers, none of the participants in business or Government employment. The majority of the participants, 49.8% (n=126), live in families with 4 to 5 members, 36.8% (n=93) have 2 to 3 members in their household and 13.4% (n=34) in families of 6 to 8 members there were no families with more than 10 members, in the type of family 57.3% (n=145) belongs to joint families and 42.7% (n=108) belongs to nuclear families. cardiopulmonary resuscitation only 7.1% (n=18) of participants had heard of cardiopulmonary resuscitation, while the remaining 92.9% (n=225) were not aware; most participants 94.9% (n=240) gained cardiopulmonary resuscitation knowledge from multiple sources, including schools, social media and others, 4.7% (n=2) learned through Television, Radio, community health programs and 0.4% (n=1) gained cardiopulmonary resuscitation knowledge from workplace training or family and friends. Table 2 shows the assessment level of knowledge, gaps in knowledge and misconceptions related to cardiopulmonary resuscitation among Adults (n=253). In this medium, the score for knowledge about cardiopulmonary resuscitation is 1.0, with a mean score of 1.35 and a Standard Deviation of 1.66. In the cardiopulmonary resuscitation practice knowledge, the median score is 0.0, with a Mean of 1.26 and a Standard Deviation of 1.99. In the willingness and confidence in performing cardiopulmonary resuscitation, participants showed low willingness and enthusiasm, with a Median score of 0.0, the Mean of 0.34 and Standard Deviation of 0.86, the Median score of common misconception and knowledge gaps were 0.0, with a Mean of 0.33 and a Standard deviation of 0.69, indicating widespread misconception or lack of understanding and in the suggestion for cardiopulmonary resuscitation education, the Median score of 1.0 a Mean of 3.62 and a Standard Deviation of 4.81, in the last overall knowledge score was 1.0 with a Mean of 3.52 and Standard Deviation of 4.66 (Figure 1 - see PDF) Table 3 show frequency and percentage distribution level of knowledge, gaps in knowledge and misconceptions related to cardiopulmonary resuscitation among Adults (n=253), from this table inadequate knowledge level (0-9). A significant majority of participants, 81.4% (n=206), have insufficient knowledge regarding cardiopulmonary resuscitation. This indicates that most adults possess minimal understanding with considerable gaps and misconceptions about the practice of cardiopulmonary resuscitation. In the moderately adequate knowledge (10-19), only 18.6% (n=47) of the participants fall into the category of moderately insufficient knowledge and these adults have some understanding of cardiopulmonary resuscitation, but their knowledge still requires improvement to perform the procedure or understand it fully effectively, none of the participants achieved an adequate level of knowledge, defined by highest possible score range (20-25) This underscores the overall lack of comprehensive cardiopulmonary resuscitation knowledge among the group. The data reveals a widespread level of cardiopulmonary resuscitation knowledge, with the vast majority (81.4%) scoring in the inadequate range, which suggests significance. Table 4 (Figure 2 - see PDF): Present the association between the level of knowledge, gaps and misconceptions related to cardiopulmonary resuscitation among adults with various demographic variables. The chi-square test was used to analyse the associations with a significant set at P < 0.05. The distribution of knowledge level across the age group showed no significant association with cardiopulmonary resuscitation knowledge; participants aged 21-30 years had the highest percentage of inadequate knowledge (32%) and 18-20 years (24%); despite these differences, the chi-square test result P=0.817 indicates age is not significant, significantly associated with the cardiopulmonary resuscitation knowledge. Meanwhile, the same non-significant level was found in the sexes in males (27.3%) and females (54.2%). However, the P value (P=0.836), Religion the Hindu represented largest group with inadequate knowledge (62.1%), Muslims (11.9%), Christians (6.3%) the P value (P=0.225), in number of family members most of Adult family 4-5 members (40.7%) had inadequate knowledge (E=0.949) and type of family the majority of participants in joint family (47%) and Nuclear family (34.4%) between family size not significant (p=0.759) only in the education level there was significant association between education level and cardiopulmonary resuscitation knowledge (p = 0.032) participants with no formal cardiopulmonary resuscitation education (4.3%) or primary education (40.7%) mostly had inadequate knowledge where those with higher secondary education (80.7%) were more likely to have education (8.7%) were more likely to have moderately adequate knowledge, Education plays a critical role in determining cardiopulmonary resuscitation knowledge. Although the majority of those who had never heard of cardiopulmonary resuscitation (75.5%), p=0.836 had inadequate knowledge levels, not significant most participants who reported learning about cardiopulmonary resuscitation from various sources (77.1%) had insufficient knowledge, no significant association with knowledge levels ( p = 0.877). Among the demographic variables assessed, education level was the only factor significantly associated with cardiopulmonary resuscitation knowledge, indicating that individuals with higher educational attainment tend to understand cardiopulmonary resuscitation better. Other variables such as age, sex, occupation and family structure showed no significant association, understanding the need for broader educational interventions across all demographic variables.

## Discussion:

The findings of this study revealed a poor level of cardiopulmonary resuscitation knowledge among adults residing in Kempapura, with significant knowledge gaps and misconceptions even among those with prior training. This aligns with the findings of Joseph *et al.* [8], which identified inadequate cardiopulmonary resuscitation knowledge in rural populations due to limited access to training and low awareness. Furthermore, Agarwal *et al.* [7] highlighted that despite healthcare workers' relatively higher exposure, consistent refresher courses are essential for skill retention, emphasizing the broader need for sustained education efforts across all population groups. Practical training plays a crucial role in addressing these knowledge gaps. Aqel *et al.* [6] demonstrated that hands-on cardiopulmonary resuscitation training improves skill acquisition and retention, particularly when combined with interactive teaching methods. However, the current study found that even those who had attended cardiopulmonary resuscitation training exhibited significant misconceptions, underlining the need for more effective and comprehensive training methodologies tailored to rural contexts. The association between education levels and cardiopulmonary resuscitation knowledge observed in this study echoes the findings of Pareek *et al.* [9], who reported a significant correlation between educational background and awareness of life-saving techniques. Similarly, Joshi *et al.* [10] emphasized integrating cardiopulmonary resuscitation education into school curricula and community outreach programs to improve baseline knowledge. Misconceptions about chest compression techniques, cardiopulmonary resuscitation effectiveness and when to perform resuscitation remain pervasive, as noted by Becker *et al.* [11], who stressed that inadequate knowledge directly impacts bystander intervention rates. Addressing these misconceptions through targeted community-level interventions, as suggested by Agarwal *et al.* [7], could significantly enhance emergency response capabilities in rural areas. The findings underscore the urgent need for structured cardiopulmonary resuscitation training programs in rural regions in light of increasing cardiac emergencies, particularly post-COVID-19. Future research should focus on evaluating the long-term retention of cardiopulmonary resuscitation skills, exploring attitudes toward training and identifying barriers to knowledge acquisition. Such initiatives are critical for improving survival outcomes in rural populations and bridging the gap in pre-hospital emergency care. This study highlights the significant gap in cardiopulmonary resuscitation knowledge among adults in rural Bangalore, consistent with prior findings. Ramya *et al.* [12] and Cambell *et al.* [13] reported similar trends, attributing low awareness to limited access to training and healthcare resources in rural areas. Vanashree *et al.* [14] And Bhandari and Teng [15] explained that structured training programs significantly improve cardiopulmonary resuscitation knowledge and skills, reinforcing the need for targeted interventions in underserved regions. Bhandari *et al.* [16], Ranimol *et al.* (2024) [17] and Amro *et al.* [18] emphasized the life-saving potential of bystander cardiopulmonary resuscitation and the long-term benefits of community-wide training programs, which have been shown to increase survival rates in cardiac emergencies.

## Limitations:

The study is limited to a specific rural area and may not be generalisable to other regions. Self-reported data may be subject to recall or social desirability biases. The study is expected to be completed within 3 months, including the phases of preparation, data collection, analysis and report.

## Conclusion:

Current data shows a significant gap in the knowledge of cardiopulmonary resuscitation among adults in rural areas. Improving awareness and skills was crucial to saving lives, especially in areas with limited access to immediate medical care. Education and training could bridge this gap and empower rural communities to respond effectively in an emergency. Further studies are recommended to prevent the incidence of an emergency by creating awareness among people.

## Figures and Tables

**Table 1:** Frequency and percentage distribution of demographic variables of adults N = 253

**Demographic Variables**	**F**	**%**
**Age**		
18 - 20	77	30.4
21 - 30	99	39.1
31 - 40	77	30.4
**Sex**		
Male	84	33.2
Female	169	66.8
Religion		
Hindu	192	75.9
Muslim	41	16.2
Christian	17	6.7
Others	3	1.2
**Education level**		
No formal education	18	7.1
Primary education	121	47.8
Higher secondary education	113	44.7
Undergraduate degree/Postgraduate degree	1	0.4
**Occupation**		
Unemployed	33	13
Farmer	1	0.4
Labourer	2	0.8
Business	-	-
Govt. Employee	-	-
Private sector employee	117	46.2
Homemaker	100	39.5
**Household Size:(Number of family members)**		
2 to 3 members	93	36.8
4 to 5 members	126	49.8
6 to 8 members	34	13.4
More than 10 members	-	-
**Type of family**		
Joint family	145	57.3
Nuclear family	108	42.7
**Have you ever heard of CPR?**		
Yes	18	7.1
No	225	92.9
**Where did you first learn about CPR?**		
School/College/Internet/Social Media	-	-
Workplace training/Friends/Family	1	0.4
Television/Radio/Community health programs	2	4.7
All of the above	240	94.9

**Table 2:** Assessment of knowledge, gaps in knowledge and misconceptions related to cardiopulmonary resuscitation among adults N = 253

**Knowledge**	**Median**	**Mean**	**S.D**
Knowledge of CPR	1	1.35	1.66
Knowledge of practice about CPR	0	1.26	1.99
Willingness and Confidence in Performing CPR in an Emergency	0	0.34	0.86
Common Misconceptions and Gaps in Knowledge	0	0.33	0.69
Suggestion	1	3.62	4.81
Overall knowledge	1	3.52	4.66

**Table 3:** Frequency and percentage distribution of level of knowledge, gaps in knowledge and misconception related to cardiopulmonary resuscitation among adults N = 253

**Knowledge**	**Frequency**	**Percentage (%)**
Inadequate (0 - 9)	206	81.4
Moderately adequate (10 - 19)	47	18.6
Adequate (20 - 25)	-	-

**Table 4:** Association of the level of knowledge, gaps in knowledge and misconception related to cardiopulmonary resuscitation among adults with selected demographic variables N = 253

**Demographic Variables**	**Inadequate**		**Moderately Adequate**		**Chi-Square Test & p-value**
	**F**	**%**	**F**	**%**	
Age					I_2_2=0.403
18 - 20	61	24.1	16	6.3	d.f=2
21 - 30	81	32	18	7.1	P=0.817
31 - 40	64	25.3	13	5.1	N.S
Sex					I_2_2=0.043
					d.f=1
Male	69	27.3	15	5.9	P=0.836
Female	137	54.2	32	12.6	N.S
Religion					I_2_2=4.357
Hindu	157	62.1	35	13.8	d.f=3
Muslim	30	11.9	11	4.3	P=0.225
Christian	16	6.3	1	0.4	N.S
Others	3	1.2	0	0	
Education level					I_2_2=8.764
No formal education	11	4.3	8	3.2	d.f=3
Primary education	103	40.7	17	6.7	P=0.032
Higher secondary education	91	36	22	8.7	S*
Undergraduate degree/Postgraduate degree	1	0.4	0	0	
Occupation					I_2_2=1.738
Unemployed	26	10.3	7	2.8	d.f=4
Farmer	1	4	0	0	P=0.784
Laboure	1	0.4	1	0.4	N.S
Business	-	-	-	-	
Govt. Employee	-	-	-	-	
Private sector employee	96	37.9	21	8.3	
Homemaker	82	32.4	18	7.1	
Number of family members					I_2_2=0.105
2 to 3 members	76	30	17	6.7	d.f=2
4 to 5 members	103	40.7	23	9.1	P=0.949
6 to 8 members	27	10.7	7	2.8	N.S
More than 10 members	-	-	-	-	
Type of family					I_2_2=0.094
					d.f=1
Joint family	119	47	26	10.3	P=0.759
Nuclear family	87	34.4	21	8.3	N.S
Have you ever heard of CPR?					I_2_2=0.047
					d.f=1
Yes	15	5.9	3	1.2	P=0.836
No	191	75.5	44	17.4	N.S
Where did you first learn about CPR?					I_2_2=0.262
School/College/Internet/Social media	-	-	-	-	d.f=2
Workplace training/Friends/Family	1	0.4	0	0	P=0.877
Television/Radio/Community health programs	10	4	2	0.8	N.S
All of the above	195	77.1	45	17.8	
*p<0.05
S - Significant
N.S - Not Significant
